# Toward Noninvasive Diagnosis of IgA Nephropathy: A Pilot Urinary Metabolomic and Proteomic Study

**DOI:** 10.1155/2016/3650909

**Published:** 2016-10-09

**Authors:** Michaela Neprasova, Dita Maixnerova, Jan Novak, Colin Reily, Bruce A. Julian, Jan Boron, Petr Novotny, Miloslav Suchanek, Vladimir Tesar, Petr Kacer

**Affiliations:** ^1^First Faculty of Medicine, Department of Nephrology, Charles University and General University Hospital in Prague, Prague 2, Czech Republic; ^2^Departments of Microbiology and Medicine, University of Alabama at Birmingham, Birmingham, AL 35294, USA; ^3^University of Chemistry and Technology, Technická 5, 166 28 Prague 6, Czech Republic; ^4^Essence Line, Plzeňská 130/221, 150 00 Prague 5, Czech Republic

## Abstract

IgA nephropathy is diagnosed by renal biopsy, an invasive procedure with a risk of significant complications. Noninvasive approaches are needed for possible diagnostic purposes and especially for monitoring disease activity or responses to treatment. In this pilot project, we assessed the utility of urine samples as source of biomarkers of IgA nephropathy. We used spot urine specimens from 19 healthy controls, 11 patients with IgA nephropathy, and 8 renal-disease controls collected on day of renal biopsy. Urine samples were analyzed using untargeted metabolomic and targeted proteomic analyses by several experimental techniques: liquid chromatography coupled with mass spectrometry, immunomagnetic isolation of target proteins coupled with quantitation by mass spectrometry, and protein arrays. No single individual biomarker completely differentiated the three groups. Therefore, we tested the utility of several markers combined in a panel. Discriminant analysis revealed that combination of seven markers, three metabolites (dodecanal, 8-hydroxyguanosine, and leukotriene C_4_), three proteins (*α*1-antitrypsin, IgA-uromodulin complex, and galactose-deficient IgA1), and heparan sulfate, differentiated patients with IgA nephropathy from patients with other renal diseases and healthy controls. Future studies are needed to validate these preliminary findings and to determine the power of these urinary markers for assessment of responses to therapy.

## 1. Introduction

Diagnosis of many kidney diseases and assessment of the severity of renal injury currently requires a renal biopsy for examination of the histological features. Although renal biopsy entails a risk of morbidity due to bleeding complications [1, 2], it is currently the only tool for reliable diagnosis of glomerular diseases. Therefore, noninvasive tests based on analysis of serum or urine specimens are needed [3–6]. Ideally, such a test would enable monitoring of disease progression and assessment of responses to treatment [7]. Efforts in this direction have identified clinical and molecular biomarkers capable of predicting outcomes in chronic kidney diseases; yet their introduction into clinical practice remains a challenge [8–10].

In this study, we assessed the utility of several analytical techniques to identify urinary compounds that are differentially present in the urine samples from patients with IgA nephropathy (IgAN) versus renal-disease controls and healthy controls. The tested markers included urinary proteins and low-molecular-mass compounds [11], such as products of oxidative stress formed during oxidative damage of phospholipids, proteins, and nucleic acids. The panel of biomarkers was selected based on data from our mass spectrometric untargeted metabolomics profiling and targeted proteomic analysis, using available database and software solutions. Our statistical approaches using ANOVA eliminated analytes that have not statistically contributed to the separation of the groups with *p* < 0.05. The panel of biomarkers was selected from all tested analytes based on the capacity of various combinations to differentiate the tested samples into three groups: healthy controls, patients with IgAN, and disease controls.

## 2. Materials and Methods

### 2.1. Urine Samples

Spot urine samples were collected from 19 healthy controls and 19 patients with biopsy-proven IgAN or non-IgAN renal disease. Samples from the patients with kidney diseases were collected on the day of renal biopsy, before the biopsy was performed. The urine samples were stored in aliquots at −80°C until assayed. Clinical and laboratory data for patients with biopsy-proven nephropathy [IgAN, membranous nephropathy, lupus nephritis, antineutrophilic cytoplasmic antibody (ANCA) vasculitis-associated kidney disease, and diabetic nephropathy] are summarized in Table 1. The study was approved by the Ethics Committee of the General Teaching Hospital in Prague, Czech Republic. Written informed consent was obtained from all participants.

### 2.2. Determination of Protein Biomarkers: Immunomagnetic Isolation Followed by MALDI-TOF MS Analysis

#### 2.2.1. Immunomagnetic Isolation

We followed our previously published protocol [12] with the antibodies detailed below. Candidate urinary biomarkers [6, 11] were isolated by using polyclonal affinity-purified antibodies specific for human proteins IL-6, IL-8, IgA, IgA-uromodulin complex, monocyte chemotactic protein-1 (MCP-1), epidermal growth factor (EGF), *α*1-antitrypsin, LG3 fragment of endorepellin, soluble transferrin receptor, tumstatin, endostatin, and heparan sulfate (purchased from Antibody Technology Inc.; http://www.antibodies-online.com/). MALDI matrix 1,2-dimethoxy-4-hydroxycinnamic (sinapinic acid) and other chemicals were purchased from Sigma-Aldrich (St. Louis, MO, USA) and chemical solvents were purchased from Merck (Darmstadt, Germany).

*Buffers*

 
*Buffer A*: 0.1 M sodium-phosphate buffer pH 7.4.
 
*Buffer B*: phosphate-buffered saline (PBS) pH 7.4 with 0.1% (w/v) bovine serum albumin (BSA).
 
*Buffer C*: 0.2 M Tris pH 8.5 with 0.1% (w/v) BSA.
 
*Buffer D*: 100 mM glycine (pH 2.5).

Dynabeads® M-280, tosyl-activated superparamagnetic polystyrene beads coated with polyurethane, were washed twice in Buffer A to remove sodium azide (NaN_3_) using magnetic particle concentrator following the manufacturer's protocol (Thermo Fisher Scientific, Waltham, MA, USA). Antibodies listed above (500 *μ*g each) were dissolved in 100 *μ*L of Buffer A and added to 100 *μ*L suspension of Dynabeads, mixed for 1 min, followed by 24-hour incubation at 37°C with mixing. Then, the supernatant was removed and the particles were washed twice with Buffer B (500 *μ*L) at 4°C. Free tosyl groups on the beads were blocked with Buffer C (500 *μ*L; 4 h, 37°C), followed by washing with Buffer B (500 *μ*L; 5 min, 4°C).

Each individual preparation of the antibody-coated magnetic beads was resuspended in a 0.5 mL aliquot of a urine sample and incubated with shaking for 1 h at 37°C. The supernatants were then removed and the beads were washed five times with Buffer B (500 *μ*L; 4°C, 5 min, vortexing). The captured antigens were eluted with Buffer D (50 *μ*L; 4°C, 1 min, vortexing) and the beads were magnetically separated. The eluates were desalted using C_18_ ZipTip (EMD Millipore, Billerica, MA, USA) before analysis by matrix-assisted laser-desorption ionization-time-of-flight mass spectrometry (MALDI-TOF MS). The beads were washed five times with Buffer B (500 *μ*L 4°C, 5 min, vortexing) and then resuspended in 500 *μ*L of Buffer A before the next immunomagnetic separation.

#### 2.2.2. MALDI-TOF MS Analysis

MALDI-TOF MS data were acquired on Autoflex mass spectrometer (Bruker Daltonics, Germany) with MALDI sample target (600 *µ*m Chip*™*; Bruker Daltonics). Ionization was achieved by irradiation with a nitrogen laser (337 nm) operating at 4 Hz. Ions were accelerated at 20 kV with 250 ns of pulsed ion extraction delay. Each spectrum was detected in linear positive mode and externally calibrated using a mixture of peptide/protein standards. Freshly prepared 1,2-dimethoxy-4-hydroxycinnamic acid was used as matrix (10 mg/mL) in 50% acetonitrile 0.1% (v/v) of trifluoroacetic acid. The instrument's parameters and laser energy were kept constant during a series of experiments performed on the same day for the comparison of intensity values (cps).

The urinary protein candidate biomarkers were analyzed by MALDI-TOF MS using several different concentrations of samples to determine the linearity and detection limit. Standard proteins were also analyzed using several different concentrations. The protonated molecular ion peak (MH^+^) for each protein was detectable to a sub-pmol level with a signal-to-noise ratio >50. This detection limit was comparable with immunochemical assays. MALDI-TOF MS is a semiquantitative method; however, using rigorous sample preparation and the data acquisition method, the intensity of the MH^+^ peak(s) increased linearly with increasing quantities of each protein from a nanomolar to picomolar range. Therefore, in this concentration range, the protein biomarkers could be analyzed in a quantitative manner.

The identification of galactose-deficient IgA1 in each urine sample was accomplished after derivatization of the immunoaffinity-isolated IgA1. Reducing-end labeling based on hydrazone-linkage enabled derivatization of the sample directly on MALDI target plates. For quantitative evaluation of selected ion candidates, molecular ions of derivatized monomeric IgA1, an in-house-developed software tool was used. Naturally galactose-deficient IgA1 protein purified from human plasma was used as a standard for calibration. Uromodulin-IgA complex (Antibody Technology Inc.) was used for calibration of the complex of IgA with uromodulin.

### 2.3. Antibody Microarrays

Custom-designed antibody microarrays were used to confirm MS data by comparing the relative content of different analytes in the samples. Polyclonal affinity-purified antibodies specific for human proteins IL-6, IL-8, IgA-uromodulin complex, MCP-1, EGF, *α*1-antitrypsin, LG3 fragment of endorepellin, soluble transferrin receptor, tumstatin, and endostatin were obtained from Antibody Technology Inc. The antibodies were spotted on the arrays using contact printing technology (NanoPrint*™* 2, Arrayit Corp., Sunnyvale, CA, USA) under a controlled environment with advanced 3-axis linear drives, Warp2 controllers, and 0.5 *µ*m positional resolution. Each antibody was printed in triplicate. After the antibody spotting, the microarrays were blocked (5 mL blocking buffer Arrayit; 3 × 5 min, room temperature, gently shaking) and washed with washing buffer (Arrayit; 5 mL, room temperature, 3 × 2 min). The design of the assay included fluorescent detection at 540 nm, based on using urine protein samples labeled with a green fluorescent dye, Cyanine 3. Briefly, the urine samples were desalted and concentrated and then labeled using a protein-labeling kit, following the manufacturer's instructions (Arrayit). The labeled samples were then diluted with 5 mL of reaction buffer (Arrayit) and added to the corresponding compartment of a microarray reaction tray. After 1-hour binding reactions (37°C, gently shaking), microarrays were washed using four wash cycles (3 min, 4 mL wash buffer per well). All preparation steps and the reactions with fluorescent reagents were carried out in the dark to prevent photobleaching. After the final wash, the microarrays were dried and images were acquired using a laser scanner (GenePix 4000B, 540 nm; Molecular Devices, LLC, Sunnyvale, CA, USA) and analyzed using GenePix® Pro software and Acuity® Microarray Informatics Software. The raw data were processed using an open-source software Rstudio (https://www.R-project.org/) with an incorporated limma package [13].

### 2.4. Analysis of Oxidative-Stress Biomarkers by Liquid Chromatography/Tandem Mass Spectrometry

Analysis of markers of oxidative stress was performed using liquid chromatography/mass spectrometry (LC-MS) system consisting of quaternary pump, Accela 600, Accela autosampler (Thermo Fisher Scientific) linked with a triple quadrupole mass spectrometer equipped with electrospray ionization (HESI) (TSQ Vantage, Thermo Fisher Scientific). To implement multimarker screening, we performed two types of analyses, one to detect compounds containing amino group(s) and the second to detect compounds with aldehyde and carboxylic groups. These two separate analyses used different conditions of derivatization reactions (acid versus alkaline environment) and the LC conditions (different composition of the mobile phase and different chromatographic columns). For the detection, tandem mass spectrometry was used, as detailed below.

#### 2.4.1. Determination of Amino Compounds

The compounds with an amino group [(o-tyrosine (o-Tyr), 3-nitrotyrosine (3-NO_2_-Tyr), 3-chlorotyrosine (3-Cl-Tyr), 8-hydroxyguanosine (8-OHG), and 8-hydroxy-2′-deoxyguanosine (8-OHdG)] were derivatized using 3-aminopyridyl-N-hydroxysuccinimidyl carbamate (APDS). Deuterium-labeled analogues of the analytes served as standards. To 500 *μ*L of each urine sample supplemented with the deuterium-labeled standards, 450 *μ*L of borate buffer (pH 8.5) and 50 *μ*L of APDS derivatization agent (1 mg/mL, acetonitrile) were added. Derivatization reactions were carried out for 10 min at room temperature and then the samples were heated to 55°C, to decompose the excess of the derivatization agent. The sample was then subjected to liquid chromatography electrospray ionization tandem mass spectrometry (LC-ESI-MS/MS) analysis on a chromatographic column XTerra® MS (C18 50 × 1 mm × 3.5 mm) (Waters, Republic of Ireland), using an isocratic elution method with a mobile phase consisting of acetonitrile : water (60 : 40, v/v) with 0.1% formic acid. The temperature of the column was kept at 25°C and the flow rate was 150 *μ*L/min. Assay parameters were optimized for use in neutral-loss mode in the interval 250–500 Da (Q1) → 130–380 Da (Q3) (Table 2) with collision-induced dissociation (CID) energy 15 eV in the negative electrospray ionization (ESI^−^) mode.

#### 2.4.2. Determination of Aldehydes and Carboxylic Acids

Derivatization of aldehydes [n-aliphatic aldehydes (C6–C12), malondialdehyde (MDA), 4-hydroxynonenal (4-HNE), and 4-hydroxyhexenal (4-HHE)] and compounds with a carboxyl group in the structure [8-isoprostane (8-*iso*-PGF_2*α*_), cysteinyl leukotrienes C, D, and E (Cys-LTs), and leukotriene B_4_ (LTB4)] was carried out using derivatization with Girard's reagent T (GirT) in the presence of N-(3-dimethylaminopropyl)-N′-ethylcarbodiimide hydrochloride and N-hydroxysuccinimide. Deuterium-labeled analogues of the analytes served as standards. To a 100 *μ*L aliquot of each urine sample supplemented with the deuterium-labeled standards, 10 *μ*L of derivatization reagent GirT and 10 *μ*L of N-(3-dimethylaminopropyl)-N′-ethylcarbodiimide hydrochloride together with 10 *μ*L sulfo-N-hydroxysuccinimide, 10 *μ*L of 1% hydrochloric acid, and 270 *μ*L of propan-2-ol were added. Derivatization proceeded for 30 min and the derivatized sample was immediately analyzed by LC-ESI-MS/MS. The chromatographic Thermo Hypercarb column (100 × 21 mm × 5 mm) with a Hypercarb precolumn (Thermo Fisher Scientific) was used with an isocratic elution (methanol : water (40 : 60, v/v) with pH adjusted with ammonium hydroxide to pH 9). The flow rate was 150 *μ*L/min. The column was kept at constant temperature 30°C, and 10 *μ*L volume of each sample was injected. Mass spectrometer parameters were capillary voltage 3,000 V, capillary inlet temperature 300°C, HESI evaporator temperature 300°C, sheath gas (nitrogen) pressure 45 psi, and auxiliary gas (nitrogen) 10 ArbU. Measurement parameters were optimized for the use in neutral-loss mode in the interval 150–750 Da (Q1) → 91–691 Da (Q3) (Table 3) with CID energy 16.5 eV in the positive electrospray ionization (ESI^+^) mode.

### 2.5. Statistical Analyses

#### 2.5.1. ANOVA

A one-way analysis of variance (ANOVA) was used to test whether two or more means were equal and whether the value of a single variable differed significantly among two or more levels of a factor and multiple observations at each level. In this study, the factor is “disease” at three levels: (1) healthy controls, (2) IgAN, and (3) non-IgAN renal disease. ANOVA statistical analysis was performed using Excel standard procedure for all 33 markers tested.

#### 2.5.2. Cluster Analysis

Cluster analysis is an exploratory data analysis tool for identifying homogenous groups of objects called clusters [14]. Objects in a specific cluster share many characteristics but significantly differ in objects not belonging to this cluster. Each object should be characterized by the value of (experimental) variables (features, parameters). By selecting a specific clustering procedure, one determines how clusters are to be formed. There are many different clustering procedures and also many ways of classifying them. In this study, we used hierarchical agglomerative clustering. In this method, clusters are consecutively formed from objects. Initially, this type of procedure starts with each object representing an individual cluster. These clusters are then sequentially merged according to their similarity (or dissimilarity). As a measure of association between the objects (distance metrics), the* Euclidean* distance was used in this work. All calculations were done by program XLSTAT (https://www.xlstat.com/).

#### 2.5.3. Discriminant Analysis

Discriminant analysis predicts a membership in a group or category based on observed values of several continuous variables [14]. Specifically, discriminant analysis predicts a classification *X* variable (i.e., three diagnoses) based on known continuous responses *Y* (i.e., 33 biomarkers). The data for a discriminant analysis consists of a sample of observations with known group membership together with their values on the continuous variables. In this study, we used discriminant analysis with transformed variables, the so-called principal components, to reduce the dimensionality of the problem and provide a better graphical view of the output. To verify the correct discriminant function, confusion matrix has been used, which resulted in classifying each of the objects in those categories. Another result of the discriminant analysis is a so-called confusion matrix that is actually a contingency table. It is possible to estimate selectivity and specificity of the biomarker test from the confusion matrix for two diagnoses. For samples from subjects with different diagnoses, it is possible to estimate accuracy of distribution of patients into three or more groups. Our suggestion is to calculate “partial” selectivity and specificity from the confusion matrix, that is, in this study, specificity and selectivity between groups 1 versus 2, 1 versus 3, and 2 versus 3. For perfect distribution in our study, partial selectivity and/or specificity are equal to 1. Another advantage is that individual biomarker tests could have an unsatisfactory specificity (selectivity), but their combination can be satisfactory or excellent, as in our case. This is called a “synergic” effect. All calculations were done by program XLSTAT (https://www.xlstat.com/).

## 3. Results

In this pilot project, we assessed potential urinary biomarkers using a small cohort of subjects that included 19 patients with different renal diseases (IgAN, membranous nephropathy, lupus nephritis, ANCA vasculitis-associated kidney disease, and diabetic nephropathy; Table 1) and 19 healthy controls. We used three different experimental techniques (immunoaffinity-MALDI MS, protein-array, and LC-MS/MS analyses) for quantitative marker assessment. The testing of urinary samples included an untargeted analysis of low-molecular-mass metabolites using selective reaction monitoring LC-MS/MS with conditions detailed in Tables 2 and 3 and a targeted analysis of selected proteins and heparan sulfate [15–19].

Table 4 provides mean concentrations of all detected analytes in the urine samples from healthy controls (group 1, *n* = 19), patients with IgAN (group 2, *n* = 11), and patients with other kidney diseases (disease controls; group 3, *n* = 8). Analysis of variance (ANOVA) and ROC curve analysis indicated that no individual biomarker completely differentiated the three groups and, thus, we next assessed the utility of several markers combined into a panel of biomarkers.

We evaluated all variables within the three individual groups by ANOVA and found that the measured markers can be divided into two groups. The first group of markers differentiated subjects in group 1 (healthy controls) from subjects in group 2 (IgAN patients) and/or group 3 (disease controls). However, these markers did not differentiate group 2 (IgAN patients) from group 3 (disease controls) (Box 1).

**Figure 1:**
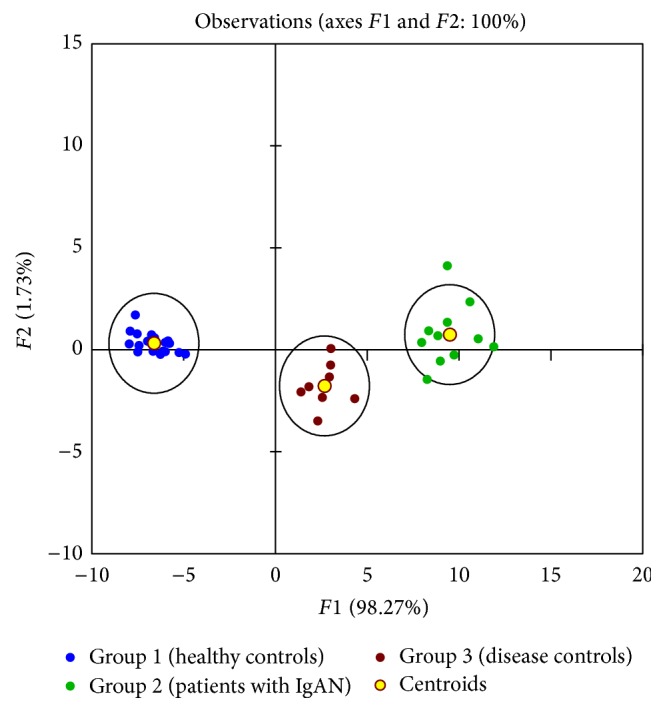
The graphical output from discriminant analysis for seven urinary markers (see also Box 2). *F*1 and *F*2, artificial axes (principal component reduction of seven selected markers into a two-dimensional space); group 1 (healthy controls, blue); group 2 (patients with IgAN, green); group 3 (disease controls, red-brown); yellow circles show centroids for each group; 95% confidential-interval ellipses are around centroids for each group.

**Figure 2:**
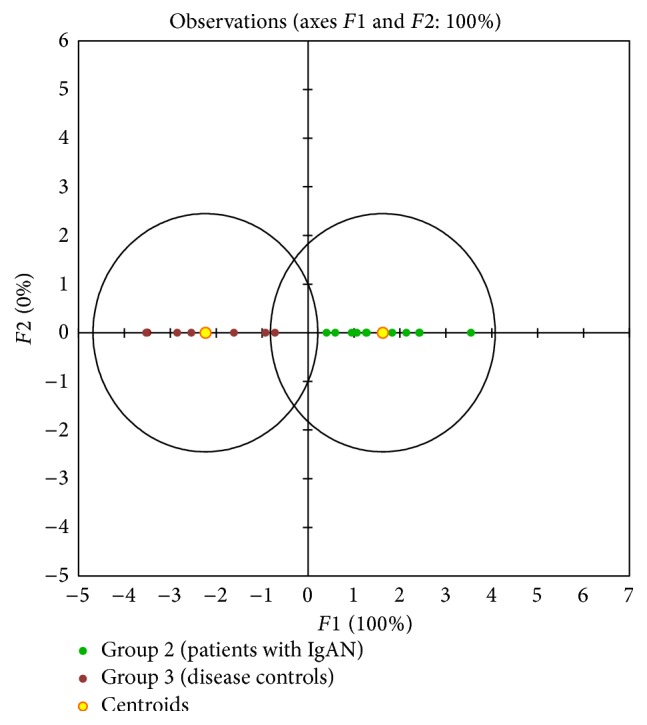
The graphical output from discriminant analysis for seven urinary markers listed in Box 2 normalized to urinary creatinine (see also Table 5) for the two groups of patients with renal disease. The same discrimination power was found for normalized markers as for nonnormalized. Group 2 (patients with IgAN, green), group 3 (disease controls, red-brown).

The second group of markers differentiated the three groups of subjects from each other. Discriminant analysis revealed that these markers included three metabolites (dodecanal, 8-hydroxyguanosine, and leukotriene C_4_), three proteins (*α*1-antitrypsin, IgA-uromodulin complex, and galactose-deficient IgA1), and heparan sulfate (Box 2, Figure 1). The conclusion on the utility of these seven markers was reproduced after normalization to urinary creatinine concentration (Table 5, Figure 2).

## 4. Discussion

Recent expansion in knowledge of the complex nature of molecular interactions has led to a better understanding of the physiological and pathological processes necessary for better diagnostic tests and treatment of various diseases. Novel and more precise analytical instruments have facilitated identifying and measuring levels of not only new individual biological markers (biomarkers) but also assessing their complex interactions that define disease pathogenesis. Molecular biomarkers are now used across many disciplines. A biomarker can be any molecule, part of a molecule, or even a particular configuration that is both detectable and measurable, where the presence, amount, or another characteristic is indicative of a particular biological state. Most diagnostic tests have been based on a single biomarker or a combination of a few biomarkers, often leading to false-positive data. To minimize this problem, multiplexing of biomarkers (i.e., signatures or panels comprised of multiple components) is now used to improve sensitivity and specificity for the diagnosis and characterization of a disease.

In our pilot study, we found that a panel of seven biomarkers (dodecanal, 8-hydroxyguanosine, leukotriene C_4_, *α*1-antitrypsin, IgA-uromodulin complex, galactose-deficient IgA1, and heparan sulfate) differentiated patients with IgAN from patients with other kidney diseases and healthy controls. However, none of the measured markers alone was specific.

Some of the seven components of our panel play a role in pathogenesis of IgAN or renal injury [16]. For example, serum levels of galactose-deficient IgA1 are elevated in many patients with IgAN [15, 20, 21]. Moldoveanu et al. [22] found elevated serum levels of galactose-deficient IgA1 in IgAN patients compared to healthy controls of Caucasian ancestry, and other studies showed similar results for patients of Asian and African-American ancestry [23]. In children with IgAN, including Caucasians and African Americans, serum levels of galactose-deficient IgA1 were elevated, but not associated with proteinuria [24]. Other investigators have shown that high serum levels of galactose-deficient IgA1 are associated with progressive loss of renal clearance function [25].

Uromodulin, also known as Tamm-Horsfall glycoprotein, is the most abundant protein in normal urine [17]. It is produced by the thick ascending limb of the loop of Henle [17] and may serve as a unique renal regulatory glycoprotein, specifically binding several cytokines, including IL-1 and TNF. Other studies found elevated levels of a fragment of uromodulin in the urine of patients with IgAN compared to that of healthy controls and patients with other glomerulonephritides [19]. A complex of uromodulin and IgA may be a diagnostic marker of IgAN; its value may be in the diagnosis of patients with an early phase of the disease with an ongoing inflammatory activity [17]. Other investigators have found increased urinary levels of complexes composed of IgA and IgG in patients with IgAN [18] or uromodulin fragments [19].

8-Hydroxyguanosine is a biomarker of nucleic acid oxidation and dodecanal is a marker of lipid peroxidation. Signs of altered oxidation have been detected in sera of patients with IgAN, including increased levels of lipoperoxide or malondialdehyde and reduced activity of superoxide dismutase, catalase, and glutathione peroxidase [26]. Recent data suggest that the nephrotoxicity of galactose-deficient IgA1-containing immune complexes in patients with IgAN is potentiated in the presence of systemic oxidation; furthermore, the intensity of the oxidative stress alters expression and progression of the disease [27].

Leukotrienes are a family of eicosanoid inflammatory mediators produced in leukocytes by the enzymatic oxidation of two essential fatty acids, arachidonic acid and eicosapentaenoic acid. The involvement of metabolites of leukotrienes in the inflammatory component of IgAN has been described [28]. Inhibitors of proliferation of mesangial cells, for example, leukotriene antagonists, may offer therapeutic options for the treatment of proliferative glomerular diseases [29].

Tubulointerstitial alterations of heparan sulfate proteoglycans that may affect inflammatory responses were observed in IgAN and other kidney diseases [30]. Other data showed increased binding of a leukocyte adhesion molecule, L-selectin, and MCP-1 to heparan sulfate proteoglycans of tubular epithelial cells in proteinuric diseases, including membranous nephropathy, IgAN, and lupus nephritis [32]. An increased expression of IL-8 on tubular epithelial cells of proteinuric patients and an increase of luminal protein may activate tubular epithelial cells to increase expression of L-selectin and MCP-1 [31]. Moreover, tubular heparan sulfate proteoglycans provide a docking platform for activation of the alternative pathway of the complement cascade via properdin, which may play a role in proteinuric renal damage [32]. Such data suggested that tubulointerstitial alterations of heparan sulfate proteoglycans in primary kidney diseases, including IgAN, may affect the inflammatory response associated with the progressive damage [31].

Elevated urinary levels of profibrotic cytokines such as IL-6, MCP-1, and soluble transferrin have been detected in patients with IgAN in contrast to the results of our study [33–35]. Another study detected elevated urinary levels of *α*1-antitrypsin in patients with IgAN, contrary to our results [36]. Urinary levels of epidermal growth factor, IL-6, and MCP-1 might act as predictor markers of renal function outcome in IgAN [37], which was not confirmed in our study.

We identified two groups of patients with different diagnoses in which individual biomarkers had unsatisfactory specificity (selectivity) but the “synergic” effect of the combination of several biomarkers was satisfactory. One of the aims of our pilot study was to suggest a new approach to test the evaluation of grouped biomarkers, implying a synergic effect of the combination of individual biomarkers to be utilized in the monitoring of disease activity and/or treatment effectiveness. This approach is also suitable for the evaluation of more diagnoses all at once.

Our preliminary data showed that combining several urinary compounds in a panel of biomarkers differentiated patients with IgAN from patients with other renal diseases and healthy controls. Further studies are needed to validate these initial findings in a larger cohort.

## 5. Conclusions

In conclusion, our pilot project found that a panel comprised of seven urinary biomarkers (8-hydroxyguanosine, dodecanal, leukotriene C_4_, *α*1-antitrypsin, IgA-uromodulin complex, galactose-deficient IgA1, and heparan sulfate) differentiated patients with IgAN from patients with other renal diseases and healthy controls. Such data need to be validated in a larger study. Moreover, a future prospective study should assess whether these biomarkers have prognostic significance and determine the power of these urinary markers for assessment of responses to therapy.

## Figures and Tables

**Box 1:**
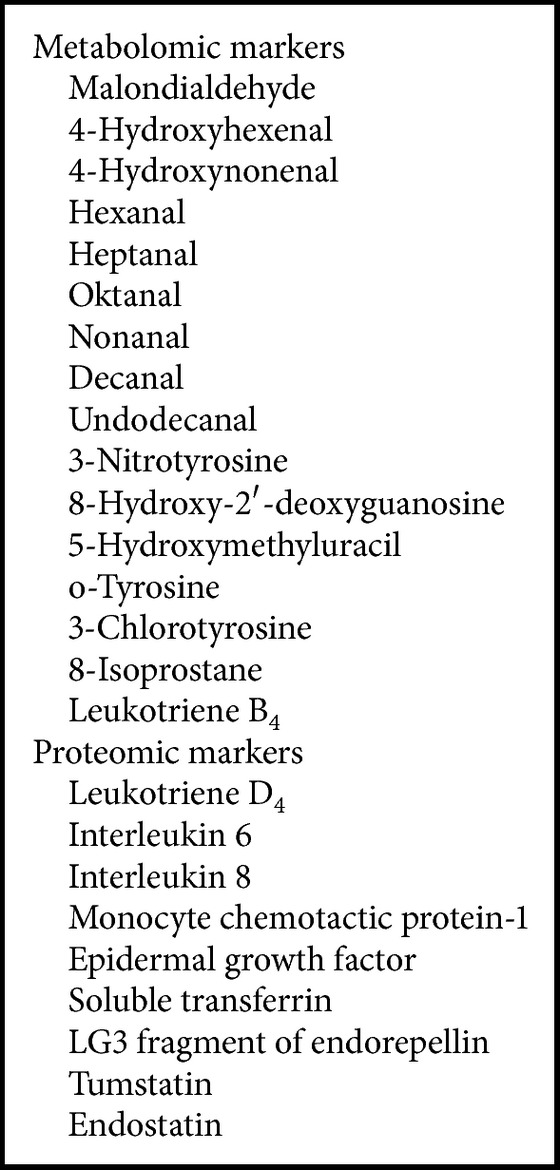
Urinary markers differentiating subjects in group 1 (healthy controls) from subjects in group 2 (IgAN patients) and/or group 3 (disease controls). These markers did not differentiate group 2 (IgAN patients) from group 3 (disease controls).

**Box 2:**
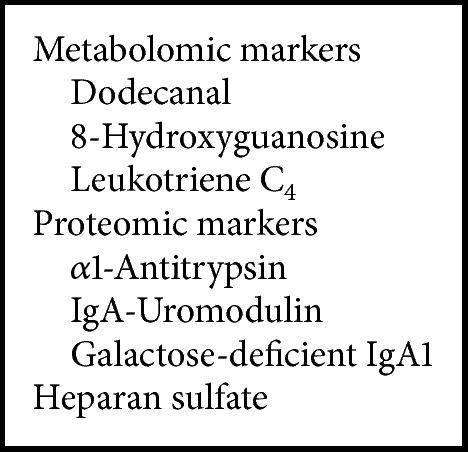
Urinary markers that differentiated all groups of subjects: group 1 (healthy controls), group 2 (IgAN patients), and group 3 (disease controls). This panel of markers differentiated the groups of subjects from each other (see also Figure 1). Discriminant analysis for this set of markers (see Table 5 for full list) confirmed the perfect distribution within the three groups of subjects.

**Table 1:** Clinical and laboratory data for patients with renal diseases.

Dg	*N*	S-urea (mmol/L)	S-Cr (*µ*mol/L)	PU (g/d)	PU (g/L)	Age (yrs)	M/F	eGFR (mL/s/1.73 m^2^)	U-Cr (mmol/L)
IgAN	11	9.8 ± 4.7	158 ± 70	1.54 ± 1.28	0.85 ± 0.61	49 ± 18	10/1	53 ± 30	7.9 ± 5.0
MN	2	10.4 ± 0.5	156 ± 4	5.92 ± 1.75	2.97 ± 1.44	56 ± 18	1/1	35 ± 6	5.7 ± 1.3
LN	2	16.1 ± 0.5	283 ± 15	3.88 ± 3.18	2.82 ± 1.43	33 ± 4	1/1	20 ± 6	7.1 ± 1.7
DN	2	29.6 ± 9.1	293 ± 19	2.24 ± 0.59	1.31 ± 0.23	62 ± 14	0/2	14 ± 1	3.6 ± 1.1
ANCA	2	17.2 ± 10.7	460 ± 68	2.86 ± 2.55	0.93 ± 0.25	42 ± 14	1/1	12 ± 5	6.2 ± 0.8

Dg, diagnosis; IgAN, IgA nephropathy; MN, membranous nephropathy; LN, lupus nephritis; DN, diabetic nephropathy; ANCA, antineutrophilic cytoplasmic antibody vasculitis-associated kidney disease; *N*, number of patients; S-urea, concentration of serum urea; S-Cr, serum creatinine; PU, proteinuria; yrs, years; M/F, male/female; eGFR, estimated glomerular filtration rate (MDRD formula); U-Cr, urinary creatinine. Data are shown as mean values ± SD.

**Table 2:** Selected reaction monitoring transitions of compounds derivatized with 3-aminopyridyl-N-hydroxysuccinimidyl carbamate reagent.

Analyte	SRM transition Q1 → Q3	CID energy [eV]
o-Tyrosine	300 → 180	15
3-Chlorotyrosine	334 → 214	16
3-Nitrotyrosine	345 → 225	14
8-Hydroxyguanosine	418 → 298	15
8-Hydroxy-2′-deoxyguanosine	402 → 282	15

Q1, precursor ion; Q3, product ion; SRM, selected reaction monitoring; CID, collision-induced dissociation.

**Table 3:** Selected reaction monitoring transitions of compounds derivatized with Girard's reagent T.

Analyte	SRM transition Q1 → Q2	CID energy [eV]
Hexanal	214 → 155	16
Heptanal	228 → 169	16
Oktanal	242 → 183	16
Nonanal	256 → 197	16
Decanal	270 → 211	16
Undecanal	284 → 225	16
Dodecanal	298 → 239	17
Malondialdehyde	169 → 101	8
4-Hydroxynonenal	273 → 214	15
4-Hydroxyhexenal	231 → 172	15
8-Isoprostane-PGF_2*α*_	471 → 412	15
Leukotriene B_4_	454 → 395	17
Leukotriene C_4_	744 → 685	14
Leukotriene D_4_	615 → 556	15
Leukotriene E_4_	558 → 499	15

Q1, precursor ion; Q3, product ion; SRM, selected reaction monitoring; CID, collision-induced dissociation; Girard's reagent T, (carboxymethyl) trimethylammonium chloride hydrazide.

**Table 4:** Mean values for all analytes in the three groups of subjects.

Analyte (^1^ng/mL, ^2^pg/mL, ^3^ *µ*g/mL)	Mean		
	Group 1	Group 2	Group 3
Metabolomic markers			
Malondialdehyde^1^	22.1	32.3	31.2
4-Hydroxyhexenal^1^	13.1	29.4	28.9
4-Hydroxynonenal^1^	23.9	33.3	35.8
Hexanal^1^	15.4	28.3	24.2
Heptanal^1^	22.5	32.1	28.9
Oktanal^1^	9.0	15.1	13.6
Nonanal^1^	12.2	15.3	14.6
Decanal^1^	9.0	13.8	12.6
Undodecanal^1^	5.6	7.2	6.9
Dodecanal^1^	6.6	8.1	7.5
3-Nitrotyrosine^2^	52.5	76.4	74.6
8-Hydroxy-2′-deoxyguanosine^2^	190.2	341.8	303.3
8-Hydroxyguanosine^2^	190.2	337.9	284.1
5-Hydroxymethyluracil^2^	90.1	168.0	156.8
o-Tyrosine^2^	54.6	91.4	82.6
3-Chlortyrosine^2^	23.8	50.2	44.5
Leukotriene B_4_^2^	151.3	381.2	293.1
8-Isoprostane^2^	20.5	43.4	38.6
Leukotriene E_4_^2^	120.0	135.5	126.0
Leukotriene D_4_^2^	55.6	65.1	62.8
Leukotriene C_4_^2^	63.9	76.4	71.6
Proteomic markers			
Interleukin 6^2^	38.8	110.5	78.8
Interleukin 8^2^	18.3	97.4	60.6
Monocyte chemotactic protein-1^1^	1.6	5.2	4.9
Epidermal growth factor^1^	5.1	2.9	3.2
*α*1-Antitrypsin^1^	16.0	55.0	40.6
IgA-Uromodulin^1^	53.8	518.0	221.9
Galactose-deficient IgA1^1^	49.8	186.7	141.3
Soluble transferrin receptor^1^	10.0	41.2	37.5
LG3 fragment of endorepellin^1^	14.0	53.2	63.9
Tumstatin^2^	72.5	158.5	167.8
Endostatin^2^	75.2	192.7	178.1
Heparan sulfate^3^	0.1	2.1	1.1

Group 1 (healthy controls), group 2 (IgAN patients), and group 3 (disease controls, patients with membranous nephropathy, lupus nephritis, antineutrophilic cytoplasmic antibody vasculitis-associated kidney disease, or diabetic nephropathy).

**Table 5:** Mean values of concentrations of measured urinary analytes normalized to urinary creatinine for patients with IgAN and disease controls.

Analyte	IgAN	Disease controls
Metabolomic markers		
Malondialdehyde (ng/mg)	6.3	6.2
4-Hydroxyhexenal (ng/mg)	5.9	5.5
4-Hydroxynonenal (ng/mg)	6.6	7.2
Hexanal (ng/mg)	5.5	4.6
Heptanal (ng/mg)	6.2	5.6
Oktanal (ng/mg)	3.0	2.6
Nonanal (ng/mg)	2.9	2.9
Decanal (ng/mg)	2.7	2.4
Undodecanal (ng/mg)	1.4	1.3
8-Hydroxyguanosine (pg/mg)	65.6	54.6
5-Hydroxymethyluracil (pg/mg)	32.6	30.2
o-Tyrosine (pg/mg)	18.1	16.0
3-Chlorotyrosine (pg/mg)	10.0	8.6
Leukotriene B_4_ (pg/mg)	75.8	56.1
8-Isoprostane (pg/mg)	8.4	7.4
Leukotriene E_4_ (pg/mg)	26.0	24.5
Leukotriene D_4_ (pg/mg)	12.4	12.3
Leukotriene C_4_ (pg/mg)	14.5	14.0
Proteomic markers		
Interleukin 6 (pg/mg)	22.4	16.0
Interleukin 8 (pg/mg)	20.1	11.5
Monocyte chemotactic protein-1 (ng/mg)	1.1	0.9
EGF (ng/mg)	0.5	0.6
*α*1-Antitrypsin (ng/mg)	10.8	7.8
IgA-Uromodulin (ng/mg)	99.1	40.3
Galactose-deficient IgA1 (ng/mg)	35.5	28.1
Soluble transferrin receptor (ng/mg)	7.8	7.1
LG3 fragment of endorepellin (ng/mg)	9.8	12.3
Tumstatin (pg/mg)	30.6	31.9
Endostatin (pg/mg)	36.4	35.7
Heparan sulfate (*μ*g/mg)	0.4	0.2
